# Valorization of *Posidonia oceanica* Biomass Extract as an Elicitor to Mitigate Aphid-Induced Stress in Sweet Pepper Plants

**DOI:** 10.3390/plants14193002

**Published:** 2025-09-28

**Authors:** Borja Ferrández-Gómez, Mar Cerdán, Juana D. Jordá, Antonio Sánchez-Sánchez

**Affiliations:** 1 Department of Biochemistry and Molecular Biology and Edaphology and Agricultural Chemistry, Faculty of Sciences, University of Alicante, 03080 Alicante, Spain; borja.ferrandez@ua.es (B.F.-G.); mar.cerdan@ua.es (M.C.); juana.jorda@ua.es (J.D.J.); 2 Research Institute CIBIO (Centro Iberoamericano de la Biodiversidad), University of Alicante, 03080 Alicante, Spain; 3 Multidisciplinary Institute for Environmental Studies “Ramón Margalef”, University of Alicante, 03080 Alicante, Spain

**Keywords:** *Posidonia oceanica*, biomass, pest, elicitor, sweet pepper, aphid

## Abstract

The increased restrictions on the use of synthetic pesticides have made the application of substances that induce plant defense mechanisms an effective alternative for protecting plants while minimizing environmental and health risks. One of the most damaging pests for sweet pepper production is the infection by the green peach aphid. This study determined the best extraction method from *Posidonia oceanica* waste and evaluated its efficacy against *Myzus persicae* (Sulzer) aphid-induced stress. In particular, the foliar application of the extract at two different doses was investigated on sweet pepper plants. The results showed that both doses decreased the contents of H_2_O_2_ (40.8% and 56.3%, respectively) and malonaldehyde (31.1% and 39.9%, respectively) in plant tissue, indicating a reduction in oxidative stress. Additionally, these elicitor extracts were effective in maintaining cell membrane integrity and photosynthetic activity. This resulted in an increase in fresh and dry weight, as well as in cellulose and hemicellulose concentration. In conclusion, *Posidonia oceanica* extracts are a promising organic farming-treatment to fight against aphid pest and its ability to stimulate plant self-defense mechanisms.

## 1. Introduction

Sweet pepper (*Capsicum spp*. L.) is one of the most popular vegetable crops because it is a source of vitamins A, C and E, as well as phenolic compounds that offer health benefits to consumers [1]. Despite its importance and popularity, sweet pepper production is adversely affected worldwide by several pests, including insects, fungi, bacteria, viruses and nematodes, which adversely affect its production worldwide [2,3]. The largest drop in production, and the higher economic losses, are caused by the green peach aphid *Myzus persicae* (Sulzer) (*M. persicae*) [4]. This pest can damage plants in a short period of time due to its proliferation rate and resistance to numerous insecticides [5].

The growing demand for organic farming production and the increasing awareness of the harmful effects of synthetic pesticides on human health and the environment have led to farmers and producers adopting alternative methods for crop protection methods against biotic stresses. These methods include the exogenous application of substances that induce defense responses in plants [6,7,8].

Plant elicitors are compounds obtained from different sources which can induce plant responses to stress, leading to increased biosynthesis of secondary plant metabolites. This process is termed as an elicitation [9]. Furthermore, an elicitor is also defined as a molecule that results in a plant response providing efficient resistance toward non-adapted pathogens [10]. Currently, the most used elicitor products are substances such as chitosan, benzoic acid, salicylic acid, jasmonic acid, and their derivatives [11]. However, in recent years, the use of plant extracts has been widely investigated due to their potential efficacy as elicitor compounds of such extracts based on their mineral content and several secondary metabolites (such as alkaloids, flavonoids, phenols, tannins, saponins, and sterols) [6,12,13]. These compounds improve plant resistance to biotic stress by altering the metabolic processes and the activity of antioxidant and antimicrobial enzymes. Furthermore, they can reduce the reactive oxygen species (ROS) generation [14,15,16,17].

Besides organic compounds, it is important to highlight the effects of nutrients on plant physiology. In this regard, mineral nutrition is essential for plant development and physiology, with calcium, iron, and magnesium standing out among the macronutrients. It should be emphasized that calcium performs structural functions in the cell wall and acts as a second messenger in signaling growth processes and stress response [18]. Iron contributes to chlorophyll synthesis and key redox reactions in photosynthesis and respiration, and is crucial for energy metabolism [19]. Magnesium, meanwhile, is the central atom of the chlorophyll molecule and is a cofactor of numerous enzymes involved in carbon fixation and energy transport [20]. Therefore, the availability of these nutrients in balance, together with other macro- and micronutrients, is crucial for maintaining cellular homeostasis and avoiding deficiencies that affect plant productivity [18,19,20].

*Posidonia oceanica* L. Delile (*P. oceanica*) is a marine plant belonging to the Posidoniaceae family that forms extensive meadows between the surface and depths of 30–40 m. It has vital ecosystem functions such as oxygenating the water, retaining and fixing sediments, preventing natural coastal erosion, and providing underwater shelter for many marine species [21,22]. However, at the end of the summer, the *P. oceanica* leaves die and release debris that accumulates in large quantities on beaches [22,23,24]. This becomes problematic in touristy coastal areas because the debris must be removed from the beaches. Currently, these remains are considered urban waste and are taken to landfills where they accumulate for years due to their slow decomposition rate [25]. For this reason, it is important to find alternative uses for this waste to avoid its accumulation, reduce management costs, and generate economic benefits from its revalorization.

Nowadays, several applications are being developed to obtain uses for this waste, including its use as an additive in plastic polymers and bitumen [26,27,28], as well as its use as an absorbent material for pollutants [29], the synthesis of biochar [30,31], and its use in the agricultural industry as a growing substrate and organic soil amendment [32,33], as well as its application in the food manufacturing industry [34]. However, the extraction of bioactive compounds and secondary metabolites from *P. oceanica* waste for the synthesis of elicitors has not yet been investigated in the agricultural sector, except for an article published by our group about the germination effect of aqueous extract from *P. oceanica* [35].

It is important to emphasize that *P. oceanica* is a lignocellulosic plant with high concentrations of micro- and macronutrients (mainly calcium and iron), silicon, and secondary metabolites, including alkaloids, amino acids, sterols, fatty acids, flavonoids, phenolic compounds (TPC), and saponins [35,36,37,38,39]. These molecules play a key role in seed and plant growth, as well as in biostimulant functions against abiotic stress such as salinity or drought. They perform active functions in photosynthesis, respiration, and plant self-defense mechanisms. Additionally, they have an ideal chemical structure for scavenging ROS [14,35,37,40,41,42]. Characterization of these extracts and their antioxidant and antifungal capacities indicates their effectiveness against various biotic stressors in crops [43,44].

Due to their resilience to different pesticides, together with the growing awareness of the potential negative effects of synthetic pesticides on human health and the environment, it is important to consider alternative products, such as plant extracts obtained from *P. oceanica* residues. There is also a need for sustainable, innovative, safe, and high-quality agricultural production. The objectives of this work were to: (i) optimize the efficiency of the extraction process from *P. oceanica* residues using three different methods, (ii) evaluate the effectiveness of foliar application of *P. oceanica* extract to prevent the harmful effects of aphid infection on sweet pepper plants, and (iii) determine the most effective dose of *P. oceanica* extract in reducing aphid damage to sweet pepper plants.

## 2. Results

### 2.1. Characterization of the Posidonia oceanica Extracts

The analysis of macro- and micronutrients, as well as the concentration of silicon (Si) and TPC of aqueous *P. oceanica* extracts obtained with the three methods are reported in Table 1. The extraction process using magnetic stirring (POE-M) was able to solubilize the highest amount of nutrients, especially for Na, K, Ca, and Mn, compared to POE-S and POE-R. However, the Na concentration (110.4 mg/L) was of quite high value, and it could promote salt stress in sweet pepper plants. Furthermore, magnetic stirring extraction had a lower yield of Si and TPC than Soxhlet extraction (POE-S) and water reflux (POE-R). The yields were 60.7% for Si and 48.6% for TPC, with respect to POE-S, and 63.3% for Si and 67.9% for TPC for POE-R, respectively.

The difference between POE-S and POE-R shows that extraction with water reflux produced better results for micronutrients but a lower concentration of most macronutrients, except K (Table 1). It is important to note, however, that the Na concentration was 64.4 and 41.4 mg/L for POE-S and POE-R, respectively. This indicates that the saline contribution will be lower with POE-R. Due to the higher concentrations of Fe, Zn, Mn, and Cu, as well as better extraction of silicon and TPC, the best method for aqueous extraction from *P. oceanica* waste was water reflux at 100 °C for 1 h (POE-R).

### 2.2. Effect of Posidonia oceanica Extracts on Aphid-Infected Sweet Pepper Plant Growth

Table 2 shows the results obtained for the parameters of plant development, growth, and photosynthetic pigment parameters in sweet pepper plants subjected to the different applied treatments. There were no significant differences in fresh weight (FW) or dry weight (DW) between the POE-R-diluted treatments and the control, indicating that this dose of the extract had no negative effect on plant development. However, application of the highest extract dose (POE-R) significantly reduced the FW and DW of the plants compared to the control. The greatest reduction in plant growth was observed in plants receiving the Control+25A treatment. This significant difference confirms that the *M. persicae* aphid infection caused biotic stress in the plants.

When the FW and DW of the aphid-infected plants (Control+25A) were compared with those of the control plants (Table 2), a 43.3% and 41.1% decrease, respectively, was observed. Regarding the plants treated with two doses of the extract (POE-R+25A and POE-R-diluted+25A), the pest had a lower impact than on the Control+25A plants. Regarding the content of chlorophylls and carotenoids (Table 2), plants treated with the POE-R-diluted product did not show significant differences in photosynthetic pigment content compared to the control group. However, plants treated with POE-R had decreased chlorophyll (24.6%) and carotenoid (22.3%) content compared to the control group, which is consistent with the loss in FW and DW. It is noteworthy that the reduction in chlorophyll and carotene content in the Control+25A (aphid infection) showed no significant difference compared to POE-R. The POE-R-diluted+25A treatment was effective in maintaining chlorophyll content in plants affected by aphid infection, though carotenoid levels remained low. Conversely, the POE-R treatment had no effect on aphid infection. Leaf chlorophyll and carotenoid concentrations showed no significant differences compared to the control+25A plants.

In addition, it is important to highlight that the attack of aphids, such as *M. persicae*, on pepper leaves causes a set of physiological and morphological responses in the plant, including loss of leaf fresh and dry weight, and reduction in chlorophyll content and leaf curling, which are closely related. Aphids feed by sucking phloem sap, continuously extracting water and nutrients, which leads to a loss of leaf weight as well as loss of turgor caused by sap extraction. To maintain its turgor and preserve more fresh weight, at least for a while, as it faces the stress caused by aphids, the leaf curls. The *P. oceanica* treatment did not completely eliminate curling, but it did reduce its severity, as shown by the increase in fresh weight values for the POE-R+25A and POER-R-diluted+25A treatments (Table 2).

Regarding changes in stem thickening under aphid infection, TGA signals at 270 °C (loss of weight associated with the ashing of cellulose and hemicellulose) and at 400 °C (loss of weight associated with the ashing of lignin) were measured (Table 3). Aphid infection (Control+25A) affected the content of cellulose, hemicellulose, and lignin compared to the control plant. However, this weight loss was counteracted by POE-R treatments.

### 2.3. Effect of Posidonia oceanica Extracts on Oxidative Stress of Sweet Pepper Plants

**Figure 1:**
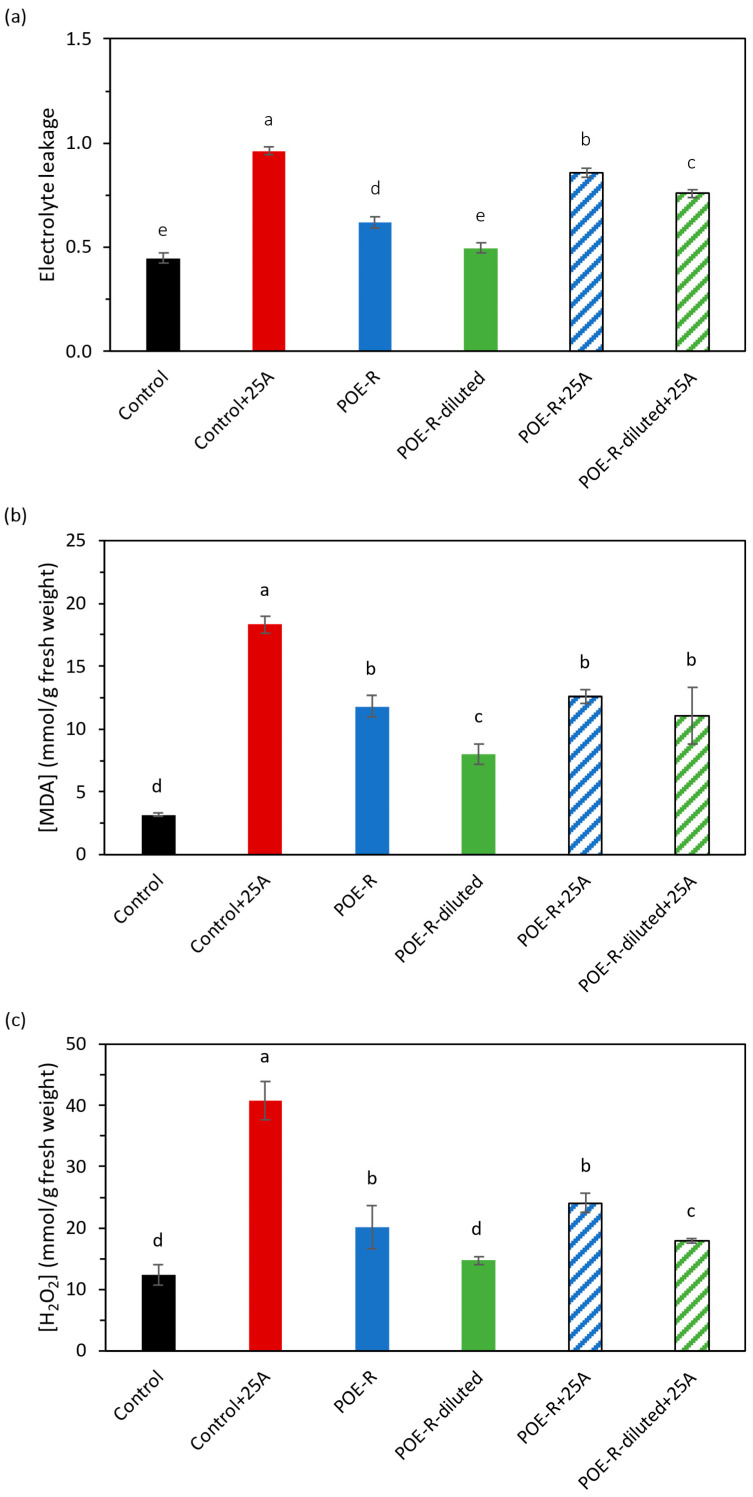
Effect of *P. oceanica* extracts by reflux method on oxidative stress in sweet pepper plant: (**a**) permeability of membrane, (**b**) MDA and (**c**) H_2_O_2_ concentration. Average with different letters is statistically different with significance *p* ≤ 0.05 according to Tukey’s test. The bars show the standard deviation of the mean (n = 8). Control: plants developed under normal conditions; Control+25A: plants infected with 25 aphids of *M. persicae* (Sulzer); POE-R: plants treated with *P. oceanica* extract from water reflux method; POE-R-diluted: plants treated with 1:1 dilution of *P. oceanica* extract from water reflux method; POE-R+25A: plants infected with 25 aphids of *M. persicae* (Sulzer) and treated with *P. oceanica* extract from water reflux method and POE-R-diluted+25A: plants infected with 25 aphids of *M. persicae* (Sulzer) and treated with 1:1 dilution of *P. oceanica* extract from water reflux method.

Several stresses in plants cause the imbalance between the formation and elimination of ROS, causing oxidative damage. Thus, determining the damage suffered in membranes because of oxidative stress is necessary to establish the permeability of the membranes by measuring the electrolyte leakage through the walls of the membranes, as well as the amount of H_2_O_2_ and MDA in plant cells. Infection with aphids in plants untreated with *P. oceanica* extracts using the reflux method resulted in a total disintegration of membrane integrity (Figure 1a) and high values for MDA (Figure 1b) and H_2_O_2_ (Figure 1c).

The pulverization of POE-R on healthy pepper plants had no effect on membrane permeability when the lowest concentrated dose was used, although a negative effect of the most concentrated extract (POE-R) was observed. These values were 86% for POE-R+25A and 76% for POE-R-diluted+25A. However, both POE-R solutions were able to reduce MDA and H_2_O_2_ levels in plants affected by aphid infection (Figure 1b,c). In any case, the control level was reached, but they reached similar levels to those of healthy plants treated with POE-R extracts. Because of the salt concentration in *P. oceanica* extracts, a parallel abiotic stress might occur. It would be related to the negative results observed until now with the most concentrated solution.

### 2.4. Effect of Posidonia oceanica Extracts on Proline Concentration of Sweet Pepper Plants

**Figure 2:**
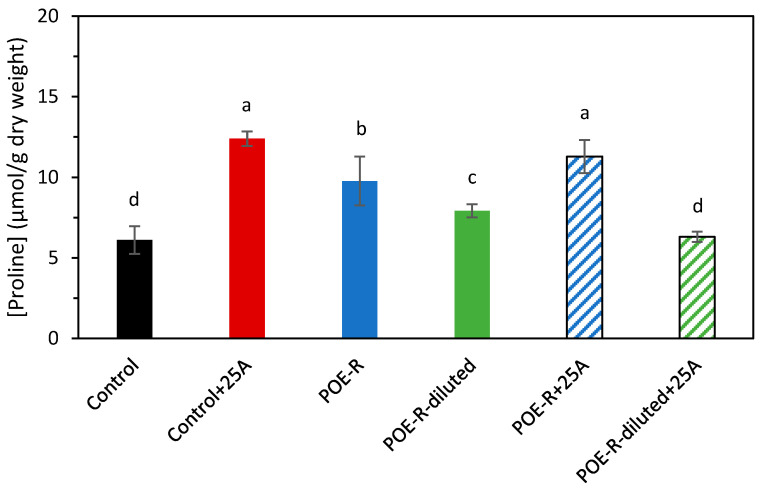
Effect of *P. oceanica* extracts by reflux method in sweet pepper plant on proline content. Average with different letters is statistically different with significance *p* ≤ 0.05 according to Tukey’s test. The bars show the standard deviation of the mean (n = 4). Control: plants developed under normal conditions; Control+25A: plants infected with 25 aphids of *M. persicae* (Sulzer); POE-R: plants treated with *P. oceanica* extract from water reflux method; POE-R-diluted: plants treated with 1:1 dilution of *P. oceanica* extract from water reflux method; POE-R+25A: plants infected with 25 aphids of *M. persicae* (Sulzer) and treated with *P. oceanica* extract from water reflux method and POE-R-diluted+25A: plants infected with 25 aphids of *M. persicae* (Sulzer) and treated with 1:1 dilution of *P. oceanica* extract from water reflux method.

Under saline or drought stress, plants generate osmolytes such as proline in order to balance osmotic pressure inside and outside of the cells. The results for proline content (Figure 2) obtained in this study agree with a saline effect due to *P. oceanica* extracts. In particular, the proline concentration in POE-R and POE-R+25A plants was, respectively, 1.6 and 1.9 times higher than in the control, with significant differences between them. As for POE-R-diluted, the proline concentration of the plants treated was slightly higher than that of the control and, for POE-R-diluted+25A sample no significant difference was observed. Thus, although foliar application of *P. oceanica* extracts could cause salt stress to the plants, mainly for the higher dose, treatment with the extract at the lower dose reduced the proline concentration in the tissue when the plant was under biotic stress caused by aphids.

### 2.5. Effect of Posidonia oceanica Extracts on Population Growth of Myzus persicae

As shown in Table 4, aqueous extracts of *P. oceanica* adversely affected the aphid life cycle. Effective fertility (M_d_) was significantly reduced by the two elicitor doses applied in this study (POE-R+1A and POE-R-diluted+1A), especially by POE-R. The preventive application of both doses of elicitor was also able to reduce the intrinsic growth rate (R_m_), although only POE-R+1A treatment produced a statistically significant reduction in the parameter (19%), with respect to the value obtained for untreated control plants (Control+1A) (Table 4). Nevertheless, a slight decrease in this parameter was also observed for the POE-R-diluted+1A treatment, although it was not statistically significant.

In relation to the pre-reproductive period (d), no statistically significant differences were found between the POE-R+1A and POE-R-diluted+1A and the Control+1A (Table 4).

## 3. Discussion

The results of this work indicate that the application of *P. oceanica* extracts to healthy plants produced an increase in proline concentration, particularly when POE-R is applied. This suggests that the high salt content in the extract, mainly sodium, may have caused salinity stress in the plants. However, this effect was mitigated by diluting the extract; visual symptoms of stress were not observed in POE-R-diluted plants. This abiotic stress can induce different actions in the plants, including a reduction in photosynthetic activity and a decrease in photoassimilates production [45,46]. These factors explain the reduction in the weight and the photosynthetic pigment content found in POE-R plants. Besides sodium, the high content of calcium, magnesium, and iron play an important role in plant growth under biotic stress. The concentration of these three minerals provided by *P. oceanica* extracts has promoted plant growth induction and maintained photosynthetic integrity [19,20]. Furthermore, their adequate availability may have helped to counteract the oxidative damage caused by aphids, as observed in the decrease in H_2_O_2_ and MDA concentrations.

Although *P. oceanica* extract contains significant quantities of phenolic compounds and silicon which have elicitor properties, these quantities were insufficient to produce a beneficial effect on salinity stress in healthy plants treated with POE-R extract.

Similarly, the H_2_O_2_ and MDA content accumulated in the tissues of POE-R sweet pepper plants was double that in the control plants. This behavior indicates that the plants experienced oxidative stress due to the high salt concentration [47].

Aphid infection caused oxidative stress in sweet pepper plants, resulting in an accumulation of H_2_O_2_ and MDA, as well as damage to the membrane integrity. The control treatment infected with 25 aphids presented the highest accumulation of H_2_O_2_ and MDA in plant tissues, reaching values 4 and 2.5 times higher than those of non-infected control plants, respectively. This increase in these oxidative stress indicators (H_2_O_2_ and MDA) is in agreement with a study carried out by Wei et al. [48] who analyzed the impact of *Aphis medicaginis* infection on different varieties of alfalfa. They concluded that MDA and H_2_O_2_ levels increased significantly up to three times compared to non-infected control plants. These results support the idea that infection by sucking insects, such as aphids, causes oxidative stress in plants.

In contrast to the effect of *P. oceanica* extract had on healthy plants, our results suggest that applying these extracts mitigates the detrimental effects of oxidative stress from aphid pests, where the most diluted extract was the most effective. According to Anjali et al., and Boukhari et al. [41,49] this beneficial effect is due to the composition of active compounds, such as silicon and phenolic compounds, which induce an effective plant response to *M. persicae* attack. Beneficial effects of Si spraying have been reported before at higher concentrations than in our *P. oceanica* extracts. In fact, Trejo-Téllez et al. [50] increased the biomass, soluble sugars, and chlorophyll content in sweet pepper plants treated with 125 mg/L of silicon.

The application of these extracts reduced the amount of H_2_O_2_ that damaged pigments and improved the cell membrane structure of chloroplasts in aphid-infected pepper plants. Similar results were reported by different authors, demonstrating that silicon produced an increase in the photosynthetic pigment content and improved membrane integrity in treated plants. This fact can be explained due to the effect of this element on the activity of certain antioxidant enzymes such as superoxide dismutase, catalase, peroxidases, and glutathione reductase [51,52,53,54]. In addition to the silicon mechanism, several authors have reported that phenolic compounds may be responsible for the improvement in membrane integrity, because they can modify peroxidation kinetics, enhance lipid packing, and contribute to the decrease in membrane flowability [41,55].

Conversely, the high calcium concentration in the POE-R extract may have acted as a signal to activate the sweet pepper defense mechanism, protecting them when infected. This behavior can be explained because plants infected by insects send signals through the Ca^2+^, due to the differences in the concentration of this element in the cytosol compared to other cellular organelles [56].

Therefore, when a plant is infected by a pathogen, the chlorophyll content decreases because the chloroplasts are degraded. However, Ni et al. [57] observed that aphid infection of wheat (*Diuraphis noxia*) reduced chlorophyll levels only in the damaged areas of the cereal, while in the areas unaffected by aphids, the chlorophyll content increased significantly compared to the levels of non-infected plants due to the activation of the defense mechanisms of the plant against the pest. In our work, *P. oceanica* extracts lowered the chlorophyll levels, though they remained above the control sample.

Finally, our results suggest that the preventive application of PO extract as a biostimulant on the aphid life cycle is able to reduce the growth rate of the pest (Table 4). *P. oceanica* extract treatments may have induced defense mechanisms in the plant against biotic stress, generating variations in the composition and the volatile organic compounds liberated by the plants, making them less attractive to the aphid. In addition, they may have also generated the accumulation in peeper plants of poisonous compounds for the aphid such as phytoalexins, phenolic compounds or saponins, although these substances were not tested in this research. Knowing the population parameters of the aphid species is essential to understand the preventive role before the pest appears; particularly, the nutritional status and the content of secondary metabolites present in the plant host may influence the quality of these herbivorous insects [58]. For most insect populations, the concept of R_m_ is a faithful representation of what happens in nature [59]. Its calculation provides an idea of the maximum capacity of a species to multiply and its sensitivity to environmental conditions or to the host plant. Our results were similar to those obtained by other authors for aphids in infected plants treated with silicon [60] or reported a mortality of 100% of the plague in plants infected with *M. persicae* after the application of oils extracted from *Pongamia glabrous* [61]. In both studies, the treatments were made after pest infection, i.e., with a curative target, and not to prevent aphid infection.

## 4. Materials and Methods

### 4.1. Extraction and Characterization of Extracts from Posidonia oceanica

The *P. oceanica* residues used in this study were collected from the shore of the southeastern coast of Spain (38°50′26″ N–0°06′20″ E). Prior to obtaining the extracts from the residues, *P. oceanica* leaves were sieved first at 2 mm, and then at 1 mm to remove sand, plastic, and other undesired materials. Then, the *P. oceanica* debris was washed several times with tap water and dried in an oven at 40 °C for 2 days.

To evaluate the effectiveness of the extraction process, three different methods were carried out: magnetic stirring (M), Soxhlet extractor (S), and water reflux (R). In all cases, 5 grams of *P. oceanica* were placed in a spherical flask with 100 mL of distilled water and heated for 1 h at 100 °C. After that, the samples were filtered with filter paper and stored in dark bottles at 4 °C. The resulting extracts were named POE-M, POE-S, and POE-R, respectively, and were analyzed for mineral and total phenolic compounds (Table 1). Macro- and micronutrients, as well as silicon, were analyzed using inductively coupled plasma mass spectrometry (ICP-MS, 7700×, Agilent, CA, USA). Phenolic compounds were quantified following the Folin–Ciocalteu method [62]. The extractions at the indicated process and the characterization were carried out in triplicate.

### 4.2. Green Peach Aphids

A colony of green peach aphids *M. persicae* (Sulzer) was maintained on sweet pepper (*Capsicum annuum* L. cv Barberito F1) plants in isolation cages in a cultivation chamber. The colony was established 2 months prior to the onset of experiments to ensure that aphids were suitably adapted to the sweet pepper plants. During breeding, environmental conditions in the growth chamber (MLR-352, Sanyo Electric Co Ltd., Moriguchi, Japan) were held constant with a temperature of 25 °C, a photoperiod of 16/8 h light/dark and relative humidity of 65%.

### 4.3. Plant Material, Cultural Conditions and Treatments

Sweet pepper (*Capsicum annuum* L. cv Barberito F1) seedlings were grown in pots of 250 mL capacity filled with a peat: vermiculite (1:1) mixture under controlled conditions of temperature (18 /25 °C (night/day)), 70% of relative humidity, and 16/8 h light/dark photocycle. The plants were irrigated daily with osmosis water and one pellet of N:P:K fertilizer (Fertiberia S.A., Valencia, Spain) was added at days 1 and 14 per pot to cover the nutritional needs of the plants.

Once the sweet pepper plants reached 15 cm of height, they were distributed in two modules of the experimental greenhouse at the University of Alicante (38°23′05″ N–0°30′47″ W, Spain), depending on if they would be infected by aphids or not. Those plants to be infected by aphids (36 plants) were placed in a module of the greenhouse provided with isolation cages, putting 12 plants in each cage. While the remaining 36 plants not to be infected by aphids were placed in a second one. Each module was equipped with an independent climate control system.

According to the concentration of silicon and phenolic compounds presented in Table 1, the best extraction method was water reflux. Due to the high concentration of Na (41.4 mg/L) and the phenolic compounds (0.56 mM) in the POE-R that could cause phytotoxicity when applied to young plants [63], the POE-R solution was diluted. Therefore, two doses of POE-R were evaluated to determine its elicitor effect: POE-R (unaltered POE-R) and POE-R-diluted (1:1 dilution of POE-R).

To evaluate the effectiveness of the foliar application of *P. oceanica* extract to combat the effects of aphid infection on sweet pepper plants, 12 pepper plants were randomly assigned to the different treatment groups. Five mL of the corresponding elicitor solution was sprayed once a week. This application was performed using a manual sprayer, and the study was conducted over a period of 35 days. Before each foliar treatment, the leaves were sprayed with 5 mL of 1% (*v*/*v*) wetting agent solution (SpaChem S.L., Valencia, Spain) to improve the penetrability of the active substances. It should be noted that the plants were infected 7 days after the beginning of the experiment by depositing 25 2-day-old *M. persicae* nymphs on a sweet pepper leaf. During the experiment, colonies were allowed to develop naturally on the plants.

The following treatments were established in this work:

(i) Control: plants developed under normal conditions;
(ii) Control+25A: plants infected with 25 aphids of *M. persicae* (Sulzer);
(iii) POE-R: plants treated with *P. oceanica* extract from water reflux method;
(iv) POE-R-diluted: plants treated with 1:1 (%*v*/*v*) dilution with distilled water of *P. oceanica* extract from water reflux method;
(v) POE-R+25A: plants infected with 25 aphids of *M. persicae* (Sulzer) and treated with *P. oceanica* extract from water reflux method;
(vi) POE-R-diluted+25A: plants infected with 25 aphids of *M. persicae* (Sulzer) and treated with 1:1 (%*v*/*v*) dilution with distilled water of *P. oceanica* extract from water reflux method.

### 4.4. Effect of Posidonia Oceanica Extracts on Population Growth of Mizus persicae

To assess the effect of *P. oceanica* extracts on the reproductive cycle of aphids, a second study was proposed. In this case, the treatments were as follows:

The following treatments were established in this work:

(i) Control+1A: plants infected with 1 aphid of *M. persicae* (Sulzer);
(ii) POE-R+1A: plants infected with 1 aphid of *M. persicae* (Sulzer) and treated with *P. oceanica* extract from water reflux method;
(iii) POE-R-diluted+1A: plants infected with 1 aphid of *M. persicae* (Sulzer) and treated with 1:1 (*v*/*v*) dilution with distilled water of *P. oceanica* extract from water reflux method.

Nine pepper plants were randomly assigned to each treatment. All solutions were applied once a week for 21 days and 48 h after the last pulverization of the *P. oceanica* extracts, a 2-day-old aphid was deposited on the underside of one of the leaves of the pepper plants.

The effect of the different treatments on the population growth of *M. persicae* was evaluated by calculating the pre-reproductive period (d), the effective fertility, and the intrinsic growth rate. The pre-reproductive period was established as the number of days that the aphid took to reproduce after plant infection for Control+1A, POE-R+1A and POE-R-diluted+1A treatments. Effective fertility (M_d_) was measured by counting the offspring per plant in a period of time equal to the pre-reproductive period and the intrinsic growth rate (R_m_) was obtained from the two previous parameters using Equation (1) described by Wyatt and White [64].

(1) $$R_{m}=0.74\cdot \frac{\ln\;(M_{d})}{d}$$

### 4.5. Physical Parameters of Sweet Pepper Plants

After 28 days of aphid infection, all sweet pepper plants were harvested, shoots and roots of each plant were washed with Extran^®^ detergent (Merck, Darmstadt, Germany) to eliminate dust, and possible residues of the treatments applied. Subsequently, the plants were washed several times with distilled water. Excess of water was removed with lab paper, and plants were weighed to measure their fresh weight (FW). After that, samples were dried overnight at 60 °C in an oven and weighed again (DW).

Stem hardening was measured by lignin detection through thermogravimetric (TGA) experiments using 10 mg of dry stem powder (SDT 2960 Simultaneous, TA Instruments, Delaware, NJ, USA). The thermobalance was purged for 1 h under N_2_:O_2_ (4:1) atmosphere, flow rate of 100 mL/min and then heated up to 600 °C (heating rate 10 °C/min).

### 4.6. Physiological Parameters in Sweet Pepper Plants

The photosynthetic pigments concentration in fresh leaves were measured by the method of Abadía et al. [65]. One g of fresh plant material with 0.1 g CaCO_3_ (Merck KGaA, Darmstadt, Germany) and 25 mL of methanol (Merck KGaA, Darmstadt, Germany) were left to stand for 4 h. The absorbance of the methanolic samples was measured at 663 and 645 nm using an UV-Vis spectrophotometer (JASCO V-630, Jasco Analitica S.L., Madrid, Spain).

Electrolyte leakage was calculated from electrolyte loss at the cellular level in four plants of each treatment [66]. For this purpose, several leaves were cut into segments of 1 cm length and 0.3 g of material were placed in Falcon tubes with 30 mL of distilled water, shaken vigorously for 1 min and incubated in a thermostatic bath at 30 °C for 2 h. After that, the electrical conductivity was measured (EC1). The samples were incubated for 15 min at 100 °C, and the electric conductivity was checked again (EC2).

The H_2_O_2_ content was measured by colorimetry as described in the methodology proposed by Jana and Choudhuri [67]. A total of 200 mg of leaves were taken and 3 mL of phosphate buffer (50 mM; pH 6.8, Merck KGaA, Darmstadt, Germany) was added for further homogenization. Once the extract was homogeneous, it was centrifuged at 10,000 rpm for 25 min and 3 mL of the supernatant was collected and mixed with 1 mL of 0.1% TiCl_4_ in 20% (*w*/*v*) H_2_SO_4_ (Merck KGaA, Darmstadt, Germany) and centrifuged again for 15 min at 10,000 rpm. Quantification was performed by UV-Vis spectrophotometry at an absorbance of 410 nm (JASCO V-630, Jasco Analitica S.L., Madrid, Spain).

Malondialdehyde (MDA) concentration was determined in foliar samples of four plants from each treatment following the method described by Shu et al. [68]. A total of 300 mg of leaves were homogenized in 3 mL of trichloroacetic acid (0.1% *v*/*v*, Merck KGaA, Darmstadt, Germany). Subsequently, the sample was filtered and 1 mL was taken and added to a 3·mL mixture in a reaction tube. The mixture consisted of trichloroacetic acid (10% *w*/*v*, Merck KGaA, Darmstadt, Germany) and thiobarbituric acid (0.65% *w*/*v*, Merck KGaA, Darmstadt, Germany) in the same proportion. The reaction tubes were then kept in a thermostatic bath at 95 °C for 25 min. After that, the samples were immersed in an ice bath for 10 min and were centrifuged for 25 min at 10,000 rpm. The supernatant absorbance was measured by UV-Vis spectrophotometry (JASCO V-630, Jasco Analitica S.L., Madrid, Spain) at 532 and 600 nm.

Finally, proline content was analyzed using the colorimetric procedure [69]. In brief, 100 mg of lyophilized leaves were homogenized with distilled water at 60 °C and centrifuged for 15 min at 10,000 rpm at 4 °C. Subsequently, 0.5 mL of the supernatant was collected and 2 mL of acidified ninhydrin (Merck KGaA, Darmstadt, Germany) was added. The samples were incubated for 30 min at 100 °C, followed by rapid cooling in an ice bath. Finally, 5 mL of toluene (Merck KGaA, Darmstadt, Germany) was added to the samples, which were shaken and stored in the dark at room temperature for 4 h before absorbance measurement in the spectrophotometer (JASCO V-630, Jasco Analitica S.L., Madrid, Spain) at 520 nm.

### 4.7. Statistical Analysis

Results obtained were evaluated using one-factor analysis of variance (ANOVA) with IBM^®^ SPSS^®^ software (23.0 version, IBM, New York, NY, USA). Statistically different groups were determined using Tukey’s test (*p* < 0.05). Prior to the ANOVA analysis, data were tested for normality and homogeneity of variance with a Shapiro–Wilk and Levene test, respectively. It should be noted that the determinations on fresh leaves were n = 8, while the determinations on dry leaves were n = 4.

## 5. Conclusions

According to the values shown for nutritional and phenolic compounds, it can be concluded that the most efficient method and with the lowest salinity contribution from the plant-based elicitor extract of *P. oceanica* residues was water reflux at 100 °C for 1 h.

Although the extract of *P. oceanica* extract at the highest dose showed potential as an elicitor against aphid infestation in sweet pepper plants, this effect was less in evidence than that observed with the most diluted dose, confirming its suitability for use as an inducer of the defense mechanisms in sweet pepper plants under the conditions of the study.

Clear evidence shows that the application of the most diluted dose of *P. oceanica* extract reduced oxidative stress caused by aphid infection, such as decreased H_2_O_2_ and MDA content in plant tissues and improved cell membrane integrity. These plants also exhibited higher fresh and dry weights, as well as higher cellulose and hemicellulose content and concentration of photosynthetic pigments in their tissues. This suggests that application of the most diluted *P. oceanica* extract may have had a beneficial effect on the photosynthetic activity of aphid-infected plants, maintaining it at levels similar to those of healthy plants.

Although the *P. oceanica* extract at the highest dose also showed a protective effect on pepper plants against aphid infestation, this was not as evident as that observed for the more diluted dose, because of the possible saline stress caused by this. *P. oceanica* extracts affected the reproductive cycle of the aphid and both doses were effective in inhibiting the multiplication rate of the pest, especially the most concentrated extract.

Further research is needed on the composition of *P. oceanica* extracts in order to isolate the bioactive compounds that affect the life cycle of aphids and remove those that cause saline stress.

## Figures and Tables

**Table 1:** Concentration of mineral composition and total phenolic compounds content in *P. oceanica* extracts using three different methods. Results are presented as mean (n = 3) ± standard deviation. Mean values followed by different letters indicate significant differences according to Tukey’s test (*p* < 0.05).

Parameter	*Posidonia oceanica*			Sig. ^1^
	Magnetic Stirring	Soxhlet Extraction	Water Reflux	
Na (mg/L)	110.4 ± 1.5 a	64.4 ± 0.2 b	41.4 ± 0.5 c	***
K (mg/L)	13.3 ± 1.2 a	4.8 ± 1.5 c	8.6 ± 0.7 b	***
Ca (mg/L)	100.3 ± 0.9 a	68.2 ± 0.6 b	44.7 ± 0.2 c	***
Mg (mg/L)	34.3 ± 0.4 c	51.0 ± 0.4 a	38.9 ± 0.2 b	***
Fe (mg/L)	0.23 ± 0.07 a	0.15 ± 0.03 b	0.30 ± 0.07 a	**
Zn (mg/L)	0.051 ± 0.005 a	0.036 ± 0.004 b	0.062 ± 0.06 a	**
Mn (mg/L)	0.023 ± 0.006 a	0.004 ± 0.002 b	0.013 ± 0.008 a	**
Cu (mg/L)	0.014 ± 0.003 c	0.029 ± 0.008 b	0.053 ± 0.005 a	***
Si (mM)	0.11 ± 0.02 b	0.28 ± 0.09 a	0.30 ± 0.05 a	***
TPC (mM)	0.18 ± 0.04 c	0.35 ± 0.04 b	0.56 ± 0.06 a	***

Sig. ^1^ is the statistical significance: ** (*p* < 0.01); *** (*p* < 0.001).

**Table 2:** Effect of POE-R and POE-R-diluted against aphid attack on fresh and dry weight and photosynthetic pigments of sweet pepper plants. Results are presented as mean (n = 8) ± standard deviation. Mean values followed by different letters indicate significant differences according to Tukey’s test (*p* < 0.05).

Parameter	Control	Control+25A	POE-R	POE-R-Diluted	POE-R+25A	POE-R-Diluted+25A	Sig. ^1^
Fresh weight (g)	35.1 ± 0.1 a	19.9 ± 0.6 d	29.6 ± 0.4 b	34.5 ± 0.8 a	24.7 ± 0.7 c	27 ± 2 b	***
Dry weight (g)	3.36 ± 0.04 a	1.98 ± 0.02 d	2.73 ± 0.1 b	3.38 ± 0.03 a	1.96 ± 0.03 d	2.05 ± 0.02 c	***
Total chlorophylls (mg/g FW)	1.46 ± 0.05 a	0.99 ± 0.08 c	1.10 ± 0.04 c	1.5 ± 0.1 a	1.05 ± 0.01 c	1.29 ± 0.05 b	**
Carotenoids (mg/g FW)	0.45 ± 0.03 a	0.27 ± 0.01 c	0.38 ± 0.03 b	0.41 ± 0.04 b	0.29 ± 0.07 c	0.33 ± 0.04 c	**

Sig. ^1^ is the statistical significance: ** (*p* < 0.01); *** (*p* < 0.001). Control: plants developed under normal conditions; Control+25A: plants infected with 25 aphids of *M. persicae* (Sulzer); POE-R: plants treated with *P. oceanica* extract from water reflux method; POE-R-diluted: plants treated with 1:1 dilution of *P. oceanica* extract from water reflux method; POE-R+25A: plants infected with 25 aphids of *M. persicae* (Sulzer) and treated with *P. oceanica* extract from water reflux method and POE-R-diluted+25A: plants infected with 25 aphids of *M. persicae* (Sulzer) and treated with 1:1 dilution of *P. oceanica* extract from water reflux method.

**Table 3:** Decomposition of cellulose, hemicellulose and lignin of sweet pepper plants to evaluate their efficacy against aphid infestation. Results are presented as mean (n = 8) ± standard deviation. Mean values followed by different letters indicate significant differences according to Tukey’s test (*p* < 0.05).

Weight Loss (mg/g)	Control	Control+25A	POE-R	POE-R-Diluted	POE-R+25A	POE-R-Diluted+25A	Sig. ^1^
Peak 270 °C	3.6 ± 0.1 a	2.8 ± 0.1 c	3.4 ± 0.1 ab	3.2 ± 0.2 b	3.4 ± 0.2 ab	3.4 ± 0.4 ab	***
Peak 400 °C	3.9 ± 0.1 ab	3.6 ± 0.3 b	4.1 ± 0.1 a	3.9 ± 0.1 ab	3.8 ± 0.2 b	3.7 ± 0.4 b	***

Sig. ^1^ is the statistical significance: *** (*p* < 0.001). Control: plants developed under normal conditions; Control+25A: plants infected with 25 aphids of *M. persicae* (Sulzer); POE-R: plants treated with *P. oceanica* extract from water reflux method; POE-R-diluted: plants treated with 1:1 dilution of *P. oceanica* extract from water reflux method; POE-R+25A: plants infected with 25 aphids of *M. persicae* (Sulzer) and treated with *P. oceanica* extract from water reflux method and POE-R-diluted+25A: plants infected with 25 aphids of *M. persicae* (Sulzer) and treated with 1:1 dilution of *P. oceanica* extract from water reflux method.

**Table 4:** Reproductive parameters of the aphid cycle under the different treatments studied. Results are presented as mean (n = 9) ± standard deviation. Mean values followed by different letters indicate significant differences according to Tukey’s test (*p* < 0.05).

Treatment	M_d_	*d*	R_m_
Control+1A	48 ± 4 a	6.7 ± 0.5	0.43 ± 0.03 a
POE-R+1A	31 ± 3 c	7.3 ± 0.5	0.35 ± 0.02 b
POE-R-diluted+1A	40 ± 3 b	6.8 ± 0.4	0.40 ± 0.2 a
Sig. ^1^	***	ns	***

Sig. ^1^ is the statistical significance: ns—indicates no significant differences (*p* > 0.05); *** (*p* < 0.001). Control+1A: plants infected with one aphid of *M. persicae* (Sulzer); POE-R+1A: plants infected with one aphid of *M. persicae* (Sulzer) and treated with *P. oceanica* extract from water reflux method and POE-R-diluted+1A: plants infected with one aphid of *M. persicae* (Sulzer) and treated with 1:1 dilution of *P. oceanica* extract from water reflux method.

## Data Availability

The raw data supporting the conclusions of this article will be made available by the authors on request.
